# CRISPR/Cas9-mediated knock-in of *BRCA1/*2 mutations restores response to olaparib in pancreatic cancer cell lines

**DOI:** 10.1038/s41598-023-45964-w

**Published:** 2023-10-31

**Authors:** Andréa Witz, Julie Dardare, Aurélie Francois, Marie Husson, Marie Rouyer, Jessica Demange, Jean-Louis Merlin, Pauline Gilson, Alexandre Harlé

**Affiliations:** 1 Département de Biopathologie, Institut de Cancérologie de Lorraine, Vandœuvre-lès-Nancy, France; 2 Université de Lorraine, CNRS UMR 7039 CRAN, Vandœuvre-lès-Nancy, France; 3 Département Recherche, Institut de Cancérologie de Lorraine, Vandœuvre-lès-Nancy, France

**Keywords:** Oncology, CRISPR-Cas9 genome editing, Cancer therapy

## Abstract

Pancreatic cancer is one of the most aggressive diseases with a very poor outcome. Olaparib, a PARP inhibitor, as maintenance therapy showed benefits in patients with metastatic pancreatic adenocarcinoma bearing germline *BRCA1/2* mutations. However, germline *BRCA* mutation has been described in only 4–7% of patients with pancreatic adenocarcinoma. A CRISPR/Cas9-mediated system was used to knock-in the c.763G > T p.(Glu255*) and c.2133C > A p.(Cys711*) mutations in cell lines to obtain truncated BRCA1 and BRCA2 proteins, respectively. A CRISPR/Cas9 ribonucleoprotein complex was assembled for each mutation and transfected into two pancreatic cell lines (T3M4 and Capan-2) and into a breast cancer cell lines (MCF7) as control. BRCA protein levels were significantly decreased in all BRCA-depleted cells (*P* < 0.05), proving the transfection efficiency of our CRISPR/Cas9 systems. As expected, the calculated olaparib IC50 were significantly reduced for all cell lines harbored *BRCA1* or *BRCA2* mutations compared to wild-type *BRCA1*/*2* cells (*P* < 0.01). Furthermore, we observed a higher induction of apoptosis after 72 h olaparib treatment in BRCA-depleted cells than in wild-type cells. This strategy might offer new insights into the management of patients with pancreatic cancer and open up new perspectives based on the in vivo use of CRISPR/Cas9 strategy.

## Introduction

Pancreatic Cancer has a poor prognosis and a 5-year ratio survival under 10%. Pancreatic ductal adenocarcinomas (PDAC), occurring in exocrine glands, represent 85% of all pancreatic cancer^1^. Surgery is considered as the only curative therapy but 80% of patients with PDAC are diagnosed at advanced stage and cannot undergo surgery^2^. Overall survival has been slightly improved in patients with advanced PDAC, with different validated regimens such as FOLFIRINOX protocol (5-Fluorouracil, Leucovorin, Irinotecan and Oxaliplatin)^3,4^, gemcitabine-plus-*nab*-paclitaxel^5^ and gemcitabine in monotherapy^6^. These regimens are often associated with high-grade toxicities and are sometimes ineffective given the complex and heterogeneous molecular mechanism of PDAC^7^.

More recently, the results of the phase III POLO trial (Pancreas Cancer Olaparib Ongoing, NCT02184195) have been published. This double-blind, placebo-controlled, and randomized study showed the efficacy of olaparib, a poly (ADP-ribose) polymerase (PARP) inhibitor (PARPi), as a maintenance therapy in patients with a germline *BRCA (breast cancer susceptibility gene)*-mutated metastatic pancreatic cancer and without tumor progression during at least 16 weeks of a first-line platinum-based chemotherapy. Median progression-free survival (PFS) was significantly improved with olaparib compared to placebo (7.4 months versus 3.8 months; hazard ratio 0.53; 95% CI [0.35–0.82]; *P* = 0.004) and the 2-year PFS was 22.1% for patients in the olaparib group compared to 9.6% for patients in the placebo group^8^. Quality of life in the olaparib group was also comparable to placebo group^8,9^. After this trial, olaparib has been approved in december, 2019 by the U.S. Food and Drug Administration (FDA) for this indication^10^.

Olaparib, approved for the management of *BRCA*-mutated ovarian^11^, metastatic breast cancer^12^, and prostate cancer^13^ is the first targeted therapy used in monotherapy to show efficacy for the management of patients with metastatic PDAC^8^. Presence of a deleterious *BRCA*-mutation is critical for the prescription of olaparib^14^ since its efficiency is based on the concept of synthetic lethality discovered in 2005 by Bryant et al*.*^15^ and Farmer et al*.*^16^*.* This concept requires two mechanisms to cause cell death. Indeed, PARP proteins are involved in DNA single-stranded breaks (SSBs) repair through the base excision repair (BER) pathway. The presence of PARPi results in stalling at replication fork and subsequent toxic DNA double-strand breaks (DSBs) formation. When PARP proteins are trapped by PARPi, DNA lesions can still be repaired by the homologous recombination repair (HRR) pathway via BRCA1 and BRCA2 proteins. In BRCA proficient cells, DSBs are repair and cells survive. In BRCA deficient cells, deleterious *BRCA1/2* mutations lead to a homologous recombination deficiency (HRD), resulting in cell death. Thus, inhibition of PARP proteins by PARPi and deleterious *BRCA1/2* mutations are both required to induce cytotoxicity^14,17,18^. Another possible etiology of HRD is the mutation of other homologous recombination (HR) genes, such as *ATM*, *PALB2* or *RAD51* genes family^19^ and this deficiency can lead to tumors sensitivity to PARPi^20^.

Only 10% of PDAC are considered as familial^21^ and germline *BRCA* mutations have been described in 4–7% of patients with pancreatic cancer^22^. In this study, we induced *BRCA1* and *BRCA2* mutations in PDAC cell-lines using a clustered regularly interspaced short palindromic repeats (CRISPR)/associated protein 9 (Cas9)-mediated knock-in technology. We evaluated whether the impact of this modification on olaparib sensitivity is the same as in the clinical setting, suggesting that this strategy, after in vivo translation, may ultimately increase the number of patients eligible for this treatment.

## Results

### Design of the CRISPR/Cas9 systems

To achieve the depletion of BRCA1 and BRCA2, a nonsense mutation for both gene was selected: *BRCA1* c.763G > T p.(Glu255*) (NM 007,294.4, rs80357009) located in exon 9 and *BRCA2* c.2133C > A p.(Cys711*) (NM 000059.3, rs535547513) located in exon 11. Each mutation was described as deleterious and pathogenic in the UMD BRCA share™^23^ and ClinVar database. gRNAs in rank 1 (called gRNA n°1) and in rank 2 (called gRNA n°2) for each mutation were kept among the 5 proposed by the CRISPR LIFEPIPE® tool (Table 1). We chose the two best crRNAs, those with a GC content within the range of 40–60% as recommended^24^.

**Table 1:** All guides RNA (gRNAs) designed by the CRISPR LIFEPIPE® tool and the Dharmacon CRISPR Design Tool.

Mutation	Rank	gRNA sequence	PAM	Cutoff site	Strand	CrisprScan score	GC %
*BRCA1* E255*	1	CTGAGAAGCGTGCAGCTGAG [43,094,766–43,094,785]	AGG	43,094,768	−	64	60
	2	CTCAGCTGCACGCTTCTCAG [43,094,766–43,094,785]	TGG	43,094,782	+	64	60
	3	AACTTGCATGTGGAGCCATG [43,094,710–43,094,729]	TGG	43,094,712	−	50	50
	4	GCATGAGTATTTGTGCCACA [43,094,692–43,094,711]	TGG	43,094,708	+	39	45
	5	GTTCTCATGCTGTAATGAGC [43,094,670–43,094,689]	TGG	43,094,686	+	16	45
*BRCA2* C711*	1	TGATTCTCTGTCATGCCTGC [32,336,473–32,336,492]	AGG	32,336,489	+	25	50
	2	TCTCTGTCATGCCTGCAGGA [32,336,477–32,336,496]	AGG	32,336,493	+	31	55
	3	CATGACAGAGAATCAGCTTC [32,336,468–32,336,487]	TGG	32,336,470	−	27	45
	4	ATGACAGAGAATCAGCTTCT [32,336,467–32,336,486]	GGG	32,336,469	−	44	40
	5	TGACAGAGAATCAGCTTCTG [32,336,466–32,336,485]	GGG	32,336,468	−	53	45

For each selected on-target mutation, 5 sequences of gRNAs are proposed with their PAM sequences, cutoff sites, DNA strand, and GC content. The CrisprScan Score are also given for each gRNA. This score is correlated with the gRNA activity^54^ and goes from 0 to 100.

**Figure 1:**
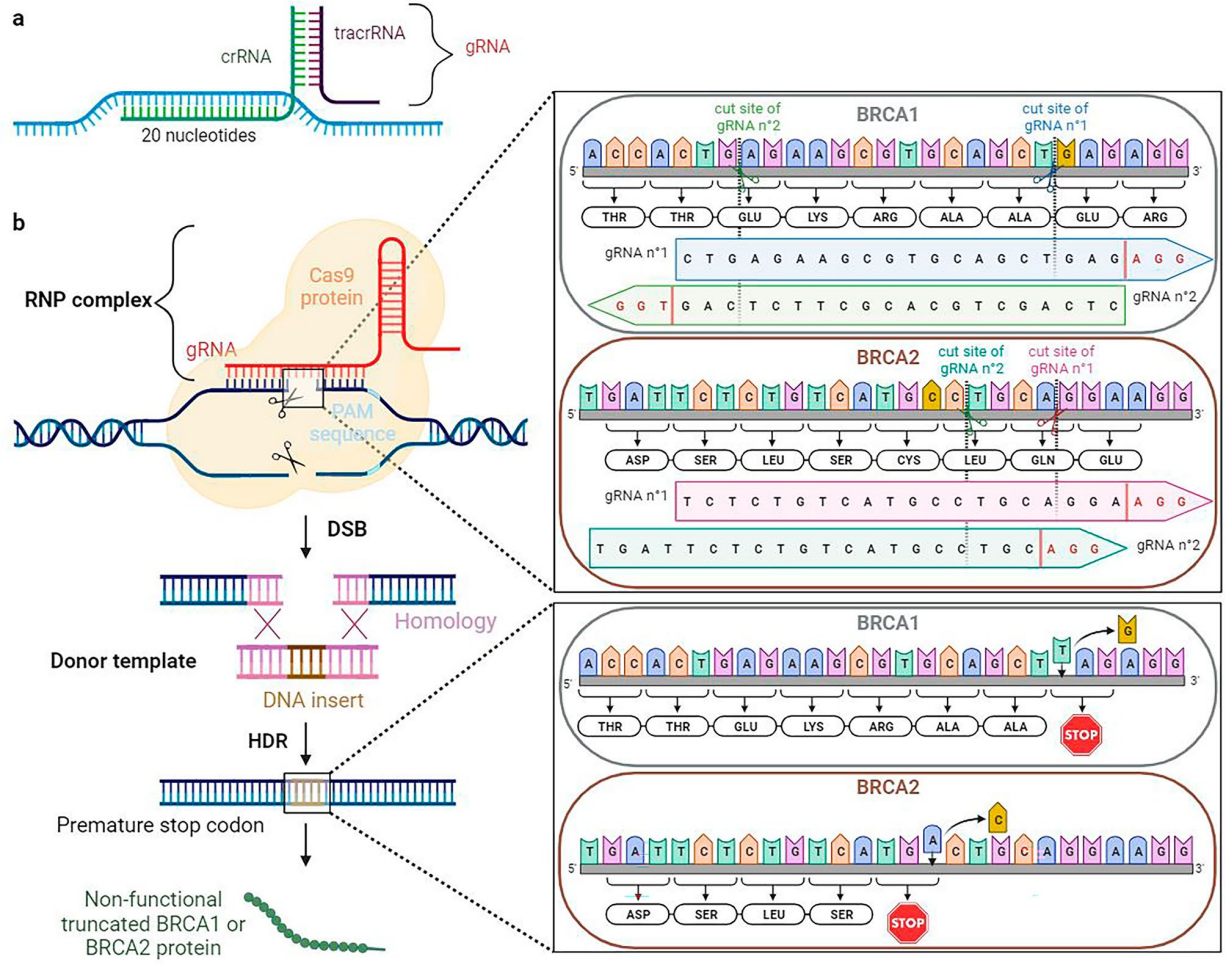
Knock-in of *BRCA1* E255* and *BRCA2* C711* mutations using a CRISPR/Cas9 RNP complex. (**a**) Guide RNA (gRNA) is composed of two elements: a trans-activating CRISPR RNA (tracrRNA) in purple and a CRISPR RNA (crRNA) in green. (**b**) A CRISPR/Cas9 RNP complex is first assembled with a gRNA and a Cas9 protein. This complex generates a double-stranded break (DSB) repaired by homology directed repair (HDR) pathway. A donor template of 61 nucleotides containing a sequence homologous to the DSB is required to introduce prematurely the termination codon and obtain a truncated nonfunctional BRCA1 or BRCA2 protein. *Created with BioRender.com.*

CRISPR/Cas9 RNP complexes were assembled with one or two gRNA for each mutation and with a single-stranded DNA (ssDNA) donor of 61 nucleotides (Fig. 1). The donor works as a template for HDR and allows the introduction of a stop codon: “TAG” for *BRCA1* and “TGA” for *BRCA2*. The recommended donor sequence for *BRCA1* c.763G > T p.(Glu255*) mutation was 5′-GATTTGAACACCACTGAGAAGCGTGCAGCT**t**agAGGCATCCAGAAAAGTATCAGGGTAGT-3′ and for *BRCA2* c.2133C > A p.(Cys711*) was 5′- TTTATTACCCCAGAAGCTGATTCTCTGTCAtg**a**CTCCAGGAAGGACAGTGTGAAAATGAT-3′.

### Knock-in of *BRCA1* E255* and *BRCA2* C711* mutations using a CRISPR/Cas9 ribonucleoprotein (RNP) complex

To verify the efficiency of our CRISPR/Cas9 systems and their capacity to induce the desired on-target mutations, we conducted a ddPCR analysis. Fractional abundances (FA) were reported in Table 2. In all tested cell lines, the on-target *BRCA1 *and *BRCA2* mutations were found for transfected cells with the CRISPR/Cas9 *BRCA1* (CC_*BRCA1*) and the CRISPR/Cas9 *BRCA2* (CC_*BRCA2*) respectively, and not for non-transfected (wild-type (WT)) and CRISPR/Cas9 control (CC_ctrl) transfected cells. Regarding MCF7 cells (not tested on PDAC cell lines), we tested the four gRNA alone and in duo. The combination of two gRNAs for the CRISPR/Cas9 RNP-assembly shows greater efficiency than gRNAs alone (FA > 90% *vs*. < 90%). For both mutations, FA is higher in MCF7 and T3M4 cells than in Capan-2 cells (up to 90%, between 50 and 80%, and about 20%, respectively). To note the experiments were carried out on the pool of transfected cells and not on clones. A cloning by limiting dilution for the three cell lines and for both mutations was attempted but failed. Indeed, after two months of culture in regular growth medium, no *BRCA1* or *BRCA2*-mutated clones survived.

**Table 2:** Fractional abundances (FA) obtained by ddPCR analysis.

Cell line	gRNA	FA after transfection (%)	FA after thawing (%)
MCF7	*BRCA1* gRNA n°1	59.40 ± 5.38	/
	*BRCA1* gRNA n°2	73.20 ± 2.14	/
	*BRCA1* gRNA n°1 + n°2	90.68 ± 0,73	4.60 ± 0.42
	*BRCA2* gRNA n°1	77.78 ± 2.64	/
	*BRCA2* gRNA n°2	82.38 ± 1.68	/
	*BRCA2* gRNA n°1 + n°2	96.58 ± 0.38	3.65 ± 0.21
Capan-2	*BRCA1* gRNA n°1 + n°2	24.85 ± 0.49	0.14 ± 0.01
	*BRCA2* gRNA n°1 + n°2	19.85 ± 2.38	0.5 ± 0.02
T3M4	*BRCA1* gRNA n°1 + n°2	54.80 ± 0.57	0.98 ± 0.34
	*BRCA2* gRNA n°1 + n°2	77.85 ± 1.63	3.04 ± 0.14

Fractional abundances (FA) are given for each pool of cells just after transfection by each CRISPR/Cas9 RNP designed and after one freeze–thaw cycle. Data are represented the mean values and standard derivation (SD) and are representative of three independent experiments.

**Figure 2:**
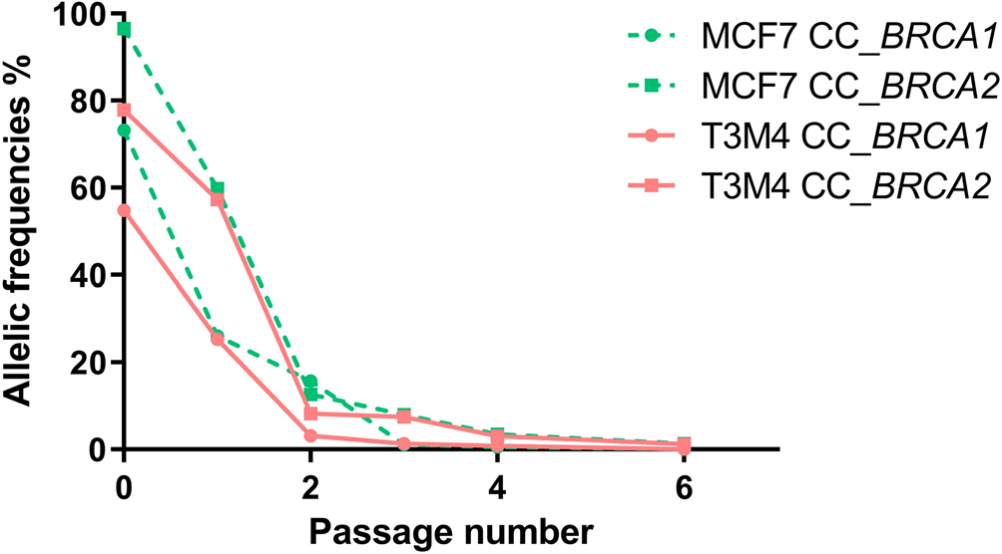
Fractional abundance according to the cell passage number. The fractional abundance (FA) of pools of transfected cells are given depending on the number of cells passaging for MCF7 and T3M4, and for both on-target mutations. No experiments were carried out on the Capan-2 cells.

Moreover, the pool of transfected cells must be used up to passage 3 maximum because the FA obtained for CC_*BRCA* transfected cells decreases by approximately 25% with each passage (Fig. 2). The same phenomenon was observed with freezing; FA of less than 4% is found for CC_BRCA transfected cells after thawing (Table 2). No experiment carried out on the Capan-2 cells due to a lower cell transfection percentage. Besides all cells keep the *BRCA2* mutation longer than the *BRCA1* mutation. Hence for each experiment, a new CRISPR/Cas9 RNP complex was assembled and transfected into all cell lines to ensure a maximal reproducibility.

### Off-target prediction of cell lines

Potential off-target sites were first predicted by the CRISPR LIFEPIPE® tool during the design of gRNA and results were confirmed by the CrispRGold tool. No off-target sites were predicted with the CRISPR LIFEPIPE® tool for all gRNAs designed. In contrast, CrispRGold predicted 3 potential off-target sites for the both gRNAs for the mutation *BRCA1* c.763G > T p.(Glu255*) (see Supplementary Table S1). Only 2 potential off-target sites were identified for the first gRNA of the mutation *BRCA2* c.2133C > A p.(Cys711*) but 13 for the second gRNAs of the mutation *BRCA2* c.2133C > A p.(Cys711*). All potential off-target sites were intronic or intergenic. The specificity score of gRNAs was on average of 6 whereas a specificity score of 12 is necessary to undoubtedly avoid off-target sites. However, no gRNA with a specificity score up or equal at 12 can be established for the two desired on-target mutations.

### Decreased BRCA protein levels in BRCA-depleted cell lines

**Figure 3:**
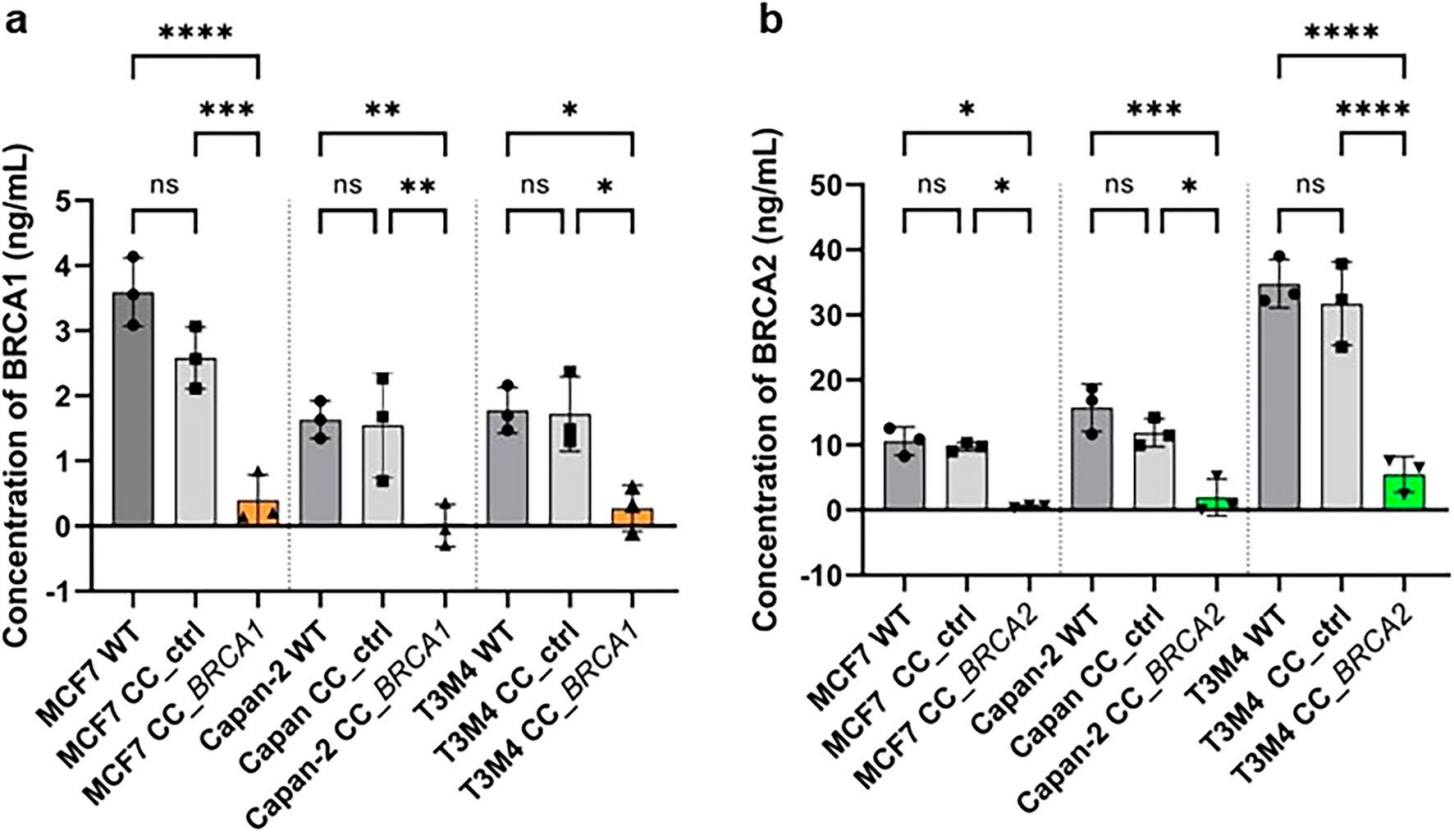
ELISA analysis of BRCA1 (**a**) and BRCA2 (**b**) proteins in MCF7, Capan-2, and T3M4 cells lysates. Data are represented as the mean values and standard derivation (SD) of 3 independent experiments for non-transfected (wild-type (WT)) cells and CRISPR/Cas9 control (CC_ctrl) (a CRISPR/Cas9 complex without donor sequences), CC_*BRCA1* and CC_*BRCA2*-transfected cells. ****P < 0.0001, ***P < 0.001, **P < 0.01, *P < 0.05 and ns not significant (ANOVA).

BRCA1 and BRCA2 protein expression levels in *BRCA* WT, BRCA-depleted and control cells were analyzed by enzyme-linked immunosorbent assays (ELISA) (Fig. 3). The level of both proteins was significantly lower in CC_*BRCA1* and CC_*BRCA2* transfected cells than in control and WT cells (*P* < 0.05) (Table 3).

**Table 3:** Summary of BRCA1 and BRCA2 proteins concentrations (ng/mL) of each cell line.

Cells	Concentration of BRCA1 proteins (ng/mL)			Concentration of BRCA2 proteins (ng/mL)		
	MCF7	Capan-2	T3M4	MCF7	Capan-2	T3M4
WT	3.60 ± 0.53	1.64 ± 0.29	1.78 ± 0.35	10.58 ± 2.17	15.73 ± 3.63	34.79 ± 3.70
CC_ctrl	2.58 ± 0.48 (ns)	1.55 ± 0.80 (ns)	1.72 ± 0.57 (ns)	9.71 ± 0.66 (ns)	11.88 ± 2.61 (ns)	31.73 ± 6.39 (ns)
CC_*BRCA*	0.40 ± 0.39 (****)	0.01 ± 0.33 (**)	0.27 ± 0.35 (*)	0.53 ± 0.20 (*)	1.93 ± 2.82 (***)	5.46 ± 2.75 (****)
*P value*	< 0.0001	0.0052	0.0105	0.0110	0.0005	< 0.0001

Data are represented as the mean values and standard derivation (SD) of 3 independent experiments for non-transfected (wild-type (WT)) cells and CRISPR/Cas9 control (CC_ctrl) (a CRISPR/Cas9 complex without donor sequences), CC_*BRCA1* and CC_*BRCA2*-transfected cells. ****P < 0.0001, ***P < 0.001, **P < 0.01, *P < 0.05 and ns not significant (ANOVA).

### Increased sensitivity of BRCA-depleted cell lines to olaparib

**Figure 4:**
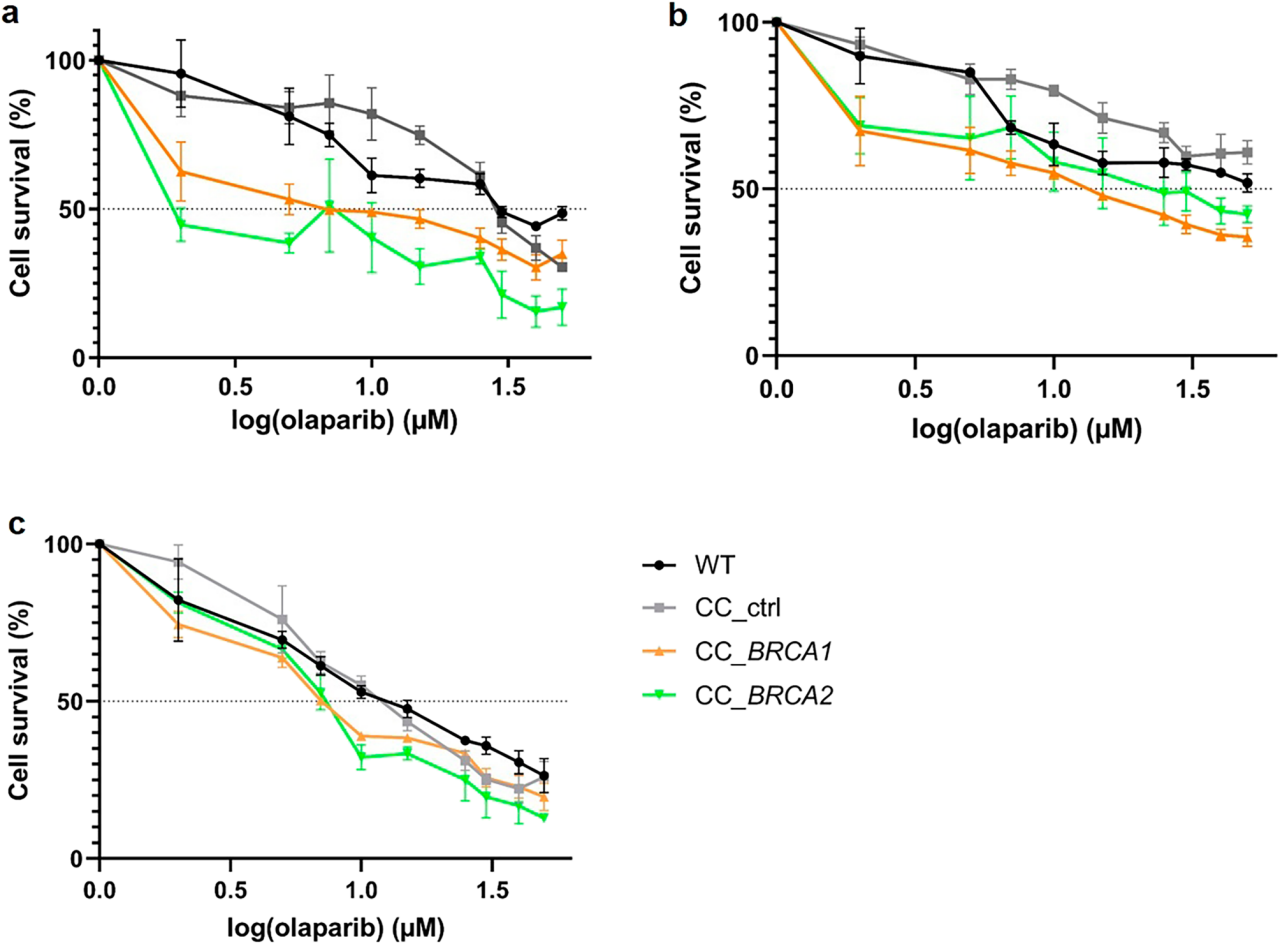
Percentage of cell viability *versus* logarithm of the concentration of olaparib obtained for non-transfected (wild-type (WT)) cells and cells transfected with a CRISPR/Cas9 control (CC_ctrl) (a CRISPR/Cas9 complex without donor sequences), a CC_*BRCA1* and a CC_*BRCA2*, for MCF7 cell line (**a**), Capan-2 cell line (**b**) and T3M4 cell line (**c**). Increasing concentrations of olaparib (0–50 µM) were applied for 72 h. Data from 3 independent experiments are expressed as mean ± SD.

The sensitivities of BRCA1 and BRCA2-depleted cells relative to control and WT cells to olaparib were analyzed with a crystal violet assay. Increasing doses of olaparib were tested and viability was evaluated 72 h later. DMSO at the concentrations used for olaparib dilutions had no impact on cell viability. Olaparib IC50 were regrouped in Table 4 and Fig. 4. No difference was observed between WT and CC_ctrl-transfected cells for all cell lines (*P* > 0.05). Induction of *BRCA1* c.763G > T p.(Glu255*) mutation and *BRCA2* c.2133C > A p.(Cys711*) mutation in MCF7 cells increased the cells sensitivity to olaparib. The olaparib IC50 was reduced from 31.73 ± 5.53 µM to 11.12 ± 4.28 µM for CC_*BRCA1*-transfected MCF7 cells (*P* < 0.01) and 5.92 ± 3.65 µM for CC_*BRCA2*-transfected MCF7 cells (*P* < 0.01). All BRCA-depleted PDAC cells were also significantly most sensitive to olaparib (*P* < 0.01) than *BRCA* WT cells. Among non-modified cell lines, Capan-2 cells were the most resistant to olaparib, whereas T3M4 cells showed greater sensitivity. In contrast, CC_*BRCA1* and CC_*BRCA2*-transfected Capan-2 cells were 3 times more sensitive to olaparib than WT Capan-2 cells, while CC_*BRCA1* and CC_*BRCA2*-transfected T3M4 cells presented an IC50 1.5 times smaller than WT T3M4 cells.

**Table 4:** Calculated IC50 (µM) of olaparib for MCF7, Capan-2 and T3M4 cell lines.

Cell lines	IC50 (µM)		
	MCF7	Capan-2	T3M4
WT	31.73 ± 5.53	> 50	13.67 ± 0.29
CC_ctrl	28.29 ± 5.65 (ns)	> 50 (ns)	12.74 ± 1.24 (ns)
CC_*BRCA1*	11.12 ± 4.28	14.19 ± 1.58	9.04 ± 0.42
*P value*	0.0036 (**)	< 0.0001 (****)	0.0011 (**)
CC_*BRCA2*	5.92 ± 3.65	17.51 ± 1.36	8.26 ± 1.12
*P value*	0.0008 (***)	< 0.0001 (****)	0.0003 (***)

Data are represented as the mean values and standard derivation (SD) of 3 independent experiments for non-transfected (wild-type (WT)) cells and CRISPR/Cas9 control (CC_ctrl), CC_*BRCA1* and CC_*BRCA2*-transfected cells. ****P < 0.0001, ***P < 0.001, **P < 0.01 and ns not significant (ANOVA).

### Increased apoptosis after treatment by olaparib for BRCA-depleted cells

**Figure 5:**
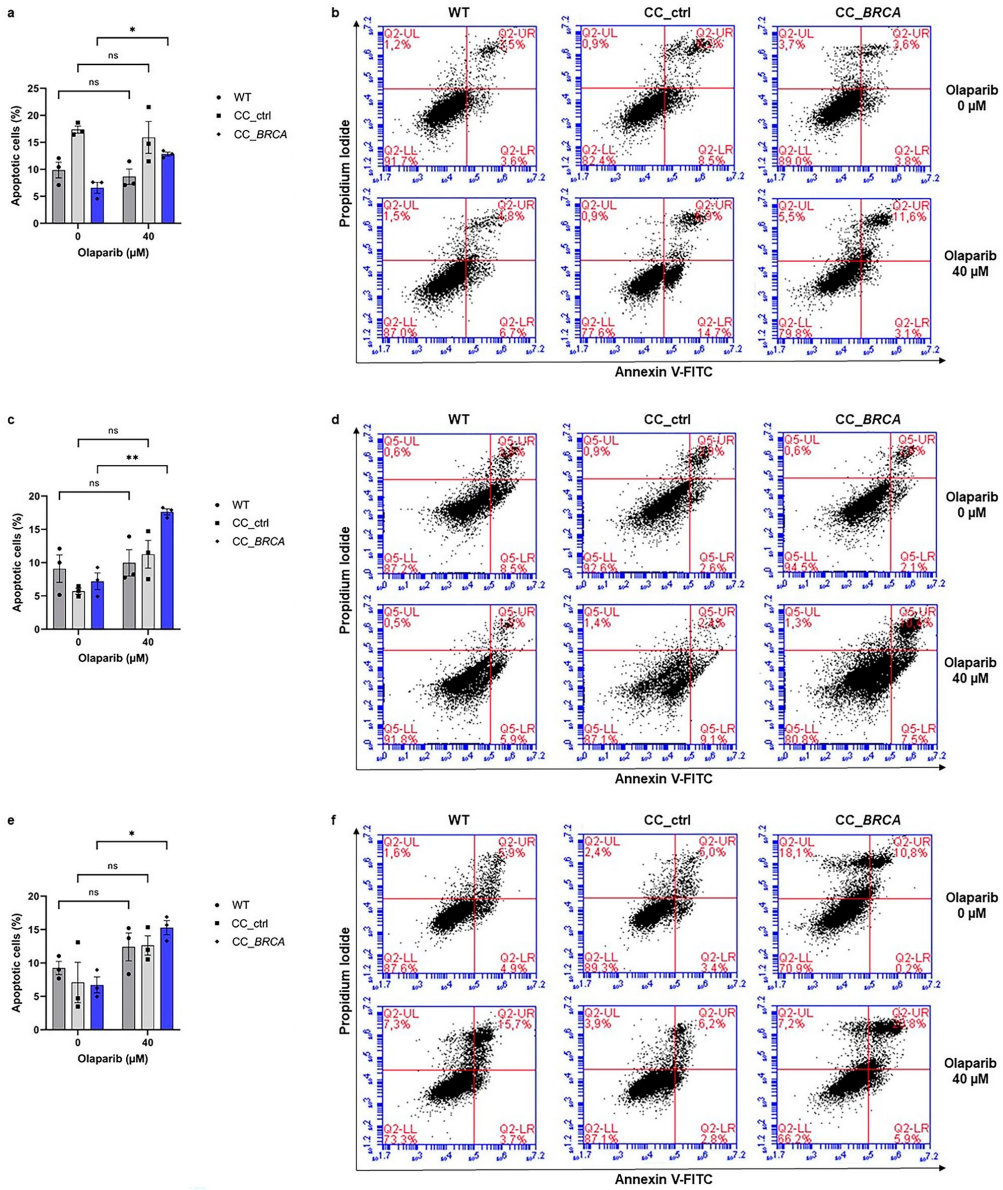
Effect of olaparib on apoptosis of MCF7 (**a**–**b**), Capan-2 (**c**–**d**), and T3M4 (**e**–**f**) cells. Non-transfected cells (wild-type (WT)) and CRISPR/Cas9 control (CC_ctrl), CC_BRCA1 and CC_BRCA2-transfected cells were treated during 72 h with olaparib at concentrations 0 and 40 µM. Apoptotic cells were detected through annexin V-FITC (AV)/propidium iodide (PI) staining and analyzed by FACS. At least 1 × 10^4^ cells/event were evaluated for each analysis. (**a**–**c**–**e**) Histograms depict the percentage of apoptotic cells. Data are represented as the mean values and SEM of 3 independent experiments. **P < 0.01, *P < 0.05 and ns not significant (Student’s t-test). (**b**–**d**–**f**) Dot plots of one representative experiment divided into four quadrants indicate necrotic (UL: AV−/PI +), late apoptotic (UR: AV + /PI +), early apoptotic (LR: AV + /PI−) and viable (LL: AV−/PI−) cells.

The sensitivity of BRCA-depleted cells to olaparib was investigated by detecting apoptotic cells labeled with Propidium Iodide (PI) and Annexin V. Cells were treated during 72 h with olaparib at 40 µM, a dose above the IC50 of the 3 models. All CC_*BRCA1* and CC_*BRCA2*-transfected cells shown an increased apoptosis after treatment by olaparib at 40 µM concentration compared to *BRCA* WT cells (Table 5 and Fig. 5). No difference was observed in the percentage of apoptotic cells after treatment with 0 µM and 40 µM of olaparib concentration for WT and control cells (*P* > 0.05).

**Table 5:** Percentage of apoptotic cells of MCF7, Capan-2 and T3M4 cell lines.

Cell lines	Apoptotic cells (%)					
	MCF7		Capan-2		T3M4	
Concentration of olaparib (µM)	0	40	0	40	0	40
WT	9.87 ± 1.46	8.685 ± 1.42 (ns)	9.09 ± 2.06	9.97 ± 1.98 (ns)	9.92 ± 1.69	12.41 ± 3.62 (ns)
CC_ctrl	17.36 ± 0.66	15.92 ± 2.96 (ns)	5.72 ± 0.45	11.25 ± 2.08 (ns)	7.07 ± 5.23	12.67 ± 2.48 (ns)
CC_*BRCA*	6.58 ± 1.02	12.81 ± 0.37	7.20 ± 1.26	17.63 ± 0.46	6.71 ± 2.05	15.29 ± 1.83
*P value*	0.0447 (*)		0.0014 (**)		0.0159 (*)	

Population of non-transfected (wild-type (WT)) cells and CRISPR/Cas9 control (CC_ctrl), CC_*BRCA1* and CC_*BRCA2*-transfected cells positive for annexin V-FITC and both for annexin V-FITC and propidium iodide was gated for apoptosis analysis after 72 h treatment with olaparib at concentrations 0 and 40 µM. At least 1 × 10^4^ cells/event were evaluated for each analysis. Data are represented as the mean values and standard error of mean (SEM) of 3 independent experiments. **P < 0.01, *P < 0.05 and ns not significant (Student’s* t*-test).

## Discussion

The PARPi olaparib is approved for the treatment of patients with breast cancer^25^ and its efficacy has been demonstrated on breast cancer cell line such as MCF7 cells^26^. The presence of a pathogenic or deleterious mutations in *BRCA1* or *BRCA2* is associated with a greater risk of developing breast, ovarian, pancreatic, and prostate cancers but are also predictive to response to olaparib. Nonetheless, a germline *BRCA* mutation is found in only in 4–7% of patients with pancreatic cancer^22^. In this study, we successfully knocked-in the *BRCA1* E255* and the *BRCA2* C711* mutations into the Capan-2 and T3M4 PDAC cell lines and in the MCF7 breast cancer cell-line, using CRISPR/Cas9-mediated technology. As previous studies have utilized a CRISPR/Cas9 system to modify MCF7 cells^27^, our initial focus was to optimize and control the effective delivery of our CRISPR/Cas9 systems into MCF7 cells prior to its application in PDAC cells. As expected, BRCA-depleted MCF7 cells exhibited higher sensitivity to olaparib than MCF7 WT cells. This result serves as control and proof of concept for the validation of our CRISPR/Cas9 system.

Our CRISPR/Cas9 system is an all-in-one HDR complex, comprising Cas9-RNP (recombinant Cas9 protein complexed with two gRNAs) and donor template DNA (ssODNs) with phosphorothioate modifications at the extremities. This all-in-one strategy increases the HDR efficiency by providing the donor template at the time of DSB occurrence^28^. Furthermore, utilization of ssODN donors along with phosphorothioate modifications at extremities greatly enhances the efficiency of HDR. The design of our ssDNA donors, as proposed by the utilized tools, was symmetric; however, study demonstrated that favoring an asymmetric donor design may facilitate HDR^29^. Therefore, further experiments using an asymmetric donor design are warranted to evaluate their potential to improve HDR efficiency.

For our all gRNAs, the DSBs induced by Cas9 and the donor template insertion-site are separated by less than ten nucleotides and all presented a PAM motif, as recommended^30^. The nature of nucleotides at position -4 from the PAM sequence also influences the editing precision. In our study, the two crRNAs for the *BRCA1* E255* mutation harbored a “T” in position -4 and for the *BRCA2* C711* mutation a “C” and “A”. The presence of a “T” or “A” at position -4 predict efficient insertions at the regions of interest. In contrast, a “G” is synonymous with a more imprecise target^31^, which concerns none of our gRNAs.

The primary limitation of CRISPR/Cas9 technology is the high probability of off-target effects, with a frequency > 50%^32^. However, reducing these off-target effects while maintaining editing efficacy remains a challenge. Several strategies have been suggested for limiting off-targets effects, including improved optimization of Cas9 and guides designs^33^. CRISPR/Cas9 design tools can now be leveraged for obtaining the best designs and identifying potential off-target sites. In our study, no off-target effect was predicted with the CRISPR LIFEPIPE® tool. In contrast, the CrispRGold tool predicted multiple off-target effects for each gRNAs, but mainly are intergenic or intronic. In cultured cells, this category of off-targets are not to be taken into account, at least for functional studies^34^. Utilizing RNP complex for delivering Cas9 protein, gRNAs and ssODN donors, as in our study, appears to be an excellent delivery method for the CRISPR/Cas9 system with decreased off-target effects and a higher rate of on-target mutations^32,33^.

The fractional abundance (FA) of CC_*BRCA*-transfected MCF7 cells is higher than FA of CC_*BRCA*-transfected PDAC cells (> 80% and < 80%, respectively) and T3M4 cells exhibit higher FA compared to Capan-2 cells (> 50% and < 20%, respectively); these mutations were not detectable in controls and non-transfected cells as expected. The differences between cell lines stem from the utilization of an identical CRISPR/Cas9 system for all of them. The DharmaFECT™ DUO, a lipid-based reagent used, has already been successfully used for the transfection of our CRISPR/Cas9 systems into MCF7 cells as reported in a previous study^35^. We optimized our transfection protocol, which involved determining the optimal incubation times and reagent concentrations, with MCF7 cells, possibly leading to the higher FA observed compared to PDAC cell lines. Aside to the transfection capacity of each cell line, the epithelial phenotype and the well-differentiated status of Capan-2 cells^36^ might explain the difficulty encountered for the transfection of these cells^37^. Furthermore, the difference between T3M4 and Capan-2 cells may be attributed to the HDR activity being restricted to the late S and G2 phases of cell cycle. The Capan-2 cells doubling time is three times longer than that of T3M4^36^, resulting in unsynchronized cell cycles that are staggered during CRISPR/Cas9 transfection. Some chemicals like nocodazole can be used to synchronize cell cycles, leading to an increased HDR. However, these molecules have potential in vivo toxicity^28^. Hence, we made the decision to not incorporate them during the CRISPR/Cas9-system optimization on cell lines with the aim of transposing the protocol in vivo more easily. In an ideal setting to increase the transfection efficiency, the CRISPR/Cas9 transfection protocol should be developed and adapted for each cell line. For simplification purposes, we utilized a universal protocol for all cell lines, leading to the assumption of a heterogeneous population produced through this approach.

Interestingly, the cell lines tested showed a decrease in FA as cells were passaged and underwent freeze–thaw cycles. We were therefore unable to generate a stable BRCA-depleted cell line, as the BRCA-depleted cells did not survive after few passages. One possible explanation is that the introduction of a loss-of-function mutation in *BRCA1* or *BRCA2* affects the cell viability of modified in vitro cells^38,39^. This phenomenon may results in the survival and expansion of only unedited cells, as estimated by their doubling times (35^40^, 60 and 23 h^36^ for MCF7, Capan-2, and T3M4 cells, respectively). After CRISPR/Cas9 transfection, cells were harvested at confluence every 4–5 days, and then either passaged or analyzed. This culture time largely exceeds the doubling time. Thus, the WT cell population expanded while the BRCA-depleted cell population remained fixed or even declined, possibly accounting for the reduction in BRCA-depleted cells after subculturing and thawing. Since we performed transient transfection using a lipid-based reagent, no stable BRCA-depleted colony was generated. To circumvent this problem, co-delivery of a CRISPR/Cas9 RNP complex and a plasmid containing a selectable marker could enrich for edited cells^41^. Moreover, plasmid-based knockout strategies can also generate stable cell lines, particularly with lentiviral transduction. However, transfection of plasmid often leads to random genome integration, host immunogenic activation in vivo, and generate potentially more off-target effects than RNP complex due to a persistent expression of Cas9 in cells^42^, even if the all-in-one plasmid circumvent some of these side effects^43^. In our study, utilization of ssODN donors and CRISPR/Cas9 RNP complex with a lipid-based transfection is advantageous in vivo but troublesome in vitro, involving CRISPR/Cas9 RNP complex transfection prior to each experiment. For example, carrying out a western blot in our study is challenging due to the transfection performed on a limited number of cells and the decreasing FA, ultimately limiting the generation of a sufficient amount of modified cells. Consequently, we performed ELISA experiments to assess the depletion of BRCA1/2 mediated by our CRISPR/Cas9 systems at the protein level. Moreover, since ddPCR assay is suitable for the detection of mutations with a very low allele frequencies^44^, we decided to use this assay to evaluate the transfection efficiency of our CRISPR/Cas9 systems. It should be noted that the FA of CC_*BRCA*-transfected MCF7 cells appears to be higher compared to those observed in other study^27^. It is probable that the presence of non-integrated donors DNA could lead to false-positive results. However, this is highly unlikely due to the large difference observed in the FA between cell lines analyzed under identical conditions (transfection time, post-transfection culture time, and day of cell harvest), as well as the larger amplicon size compared to that of the donor DNA.

Olaparib is known to be effective on cancer cells which presented a deficiency in HRR, including those with *BRCA* mutations. PARPi induce an accumulation of SSBs, which ultimately lead to DSBs and accumulation of DNA damages in HRD tumors, resulting in tumor-cell death^45^. Olaparib is the only FDA-approved PARPi in pancreatic cancer^10^, and thus is the only PARPi tested in our study. Indeed, the therapeutic efficacy of olaparib has now been proven as maintenance treatment in metastatic pancreatic cancer which carrying a germline *BRCA* mutation^8^. As expected, our study showed an anticipated increase in sensitivity to olaparib in all BRCA*-*depleted cells. Furthermore, as demonstrated in prior studies^46,47^, the treatment with 40 µM of olaparib significantly increases the apoptosis rate in CC_*BRCA1* and CC_*BRCA2*-transfected cells compared to non-transfected cells (*P* < 0.01) . Nonetheless, while the difference in sensitivity between transfected and non-transfected cells is statistically significant (*P* < 0.01), it remains small, particularly for T3M4 cells. It should be noted that we are not working with a pool of 100% mutated cells, meaning that the number of *BRCA*-mutated cells present during three days of olaparib treatment may be lower than expected. Since these cells are the most sensitive to olaparib, this may potentially alter the response to olaparib and undervalue the final outcome.

## Conclusions

In conclusion, we developed CRISPR/Cas9 systems to knock-in *BRCA1* and *BRCA2* pathogenic mutations into PDAC cell lines, resulting in a premature termination codon in each respective gene. Olaparib sensitivity was found to be increased in BRCA-depleted cells compared to *BRCA* WT cells. In addition, the Cas9-RNP used displayed short-term activity post-transfection, as its quick degradation by cellular proteases allowed for reduced number of off-target effects and transient genome editing. Therefore, this system might be easily transposed in vivo. As this technology holds the potential of reinstating PARPi sensitivity mutations, it presents an attractive therapeutic option for pancreatic cancer.

The next step in this work is to assess the in vivo translatability, consider a future clinical application. The main objective is to obtain an increase in survival rates after treatment by olaparib in in vivo models of PDAC (e.g*.* mouse models), which harbored *BRCA1/2* mutations induced by our CRISPR/Cas9 systems. The vectorization in a maximum of nucleus and the addressing only in the tumor despite a systemic introduction are the main limitations to the use of CRISPR/Cas9 technology in vivo. The delivery vehicle must be stable and versatile to allow a transient expression of the editing machinery and avoid unintentional off-target mutations within the host genome^48^. Immunogenicity and dose response must be taken into account to achieve a maximal HDR efficiency and a minimal cytotoxicity without immune response^49^. The Cas9-RNP can circumvent such issues, but its direct delivery simultaneously with the donor DNA remains a challenge in vivo. Non-viral vehicles seem to be the most suitable option for the delivering of RNP Cas9:gRNA together with the donor DNA^48^. Nanoparticles, including the CRISPR-Gold system, are good candidates for the delivery of CRISPR/Cas9 machinery. Their efficacy and in vivo safety have already been demonstrated^49–51^. Identifying off-target effects at the genomic level remains the ultimate challenge before the clinical application of the CRISPR/Cas9 system. Different methods for identifying off-target effects exist, but GUIDE-seq is the most effective genome-wide detection assay^52^. All these recommendations will allow a future clinical transposition of our CRISPR/Cas9 systems.

## Methods

### Cell lines and cell culture

Human PDAC cell line Capan-2 (RRID: CVCL_0026) and Human breast cancer cell line MCF7 (RRID: CVCL_0031), *BRCA1* and *BRCA2* wild-type (WT), were obtained from the American Type Culture Collection (Manassas, VA, USA). Human pancreatic ductal adenocarcinoma (PDAC) cell line T3M4 (RRID: CVCL_4056), *BRCA1* and *BRCA2* WT, was a gift from Dr. J. Werner (University of Heidelberg, Heidelberg, Germany)^53^. Cells were cultured in antibiotic-free RPMI 1640 (Gibco, Carlsbad, CA, USA) and incubated at 37 °C in a 5% CO_2_ humidified atmosphere. Medium was supplemented with 10% fetal bovine serum (FBS) and 2 mM L-glutamine (Sigma-Aldrich, St. Louis, MO, USA) and was changed every 3–4 days. T3M4, Capan-2 and MCF7 were periodically tested for *Mycoplasma* contamination using the VenorH GeM Mycoplasma Detection Kit (Minerva Biolabs GmbH, Berlin, Germany).

### CRISPR/Cas9-mediated knock-in

#### CRISPR RNAs (crRNAs) and homology directed repair (HDR) template sequence design

Deleterious mutations were selected to obtain truncated BRCA1 and BRCA2 proteins. Design of CRISPR RNAs (crRNAs) and donor templates was performed using the CRISPR LIFEPIPE® tool (Life&Soft, Plessis-Robinson, France) and results were confirmed using the Dharmacon CRISPR Design Tool and the Edit-R homology directed repair (HDR) Donor Designer (Dharmacon, Lafayette, CO, USA).

#### Preparation of ribonucleoprotein (RNP) complex with Cas9 protein, guide RNAs (gRNAs) and single-stranded oligodeoxynucleotides (ssODNs)

All components of ribonucleoprotein (RNP) complex were purchased from Dharmacon: Edit-R Cas9 Nuclease protein NLS (Cat #CAS11200), Edit-R synthetic trans-activating CRISPR RNA (tracrRNA) oligos (Cat #U-002005), Edit-R crRNA oligos (designed for mutations of interest) and Edit-R HDR Donor template (single-stranded oligodeoxynucleotides (ssODNs)), designed for specific knock-in of mutations of interest). tracrRNA and crRNA stock solutions were prepared according to manufacturer’s protocol « Dharmacon™ Edit-R™ synthetic guide RNA resuspension protocol ». Briefly, RNA pellets were resuspended in nuclease-free 10 mM Tris pH 7.4 (Dharmacon, Cat #B-006000-100) for a 10 µM stock solution and aliquoted to not exceed five freeze–thaw cycles. Concentrations were verified using Qubit 3.0 fluorometer (Life Technologies, Carlsbad, CA, USA) and Qubit® RNA HS Assay Kit (Life Technologies). Five nanomoles of donor oligos were resuspended in 50 µL Tris buffer to obtain a 100 µM stock solution. Concentrations were assessed using Qubit 3.0 fluorometer (Life Technologies) and Qubit® ssDNA HS Assay Kit (Life Technologies).

### Transfection

Capan-2, MCF7 and T3M4 were respectively seeded into 96-wells plates at a density of 8 × 10^3^, 1 × 10^4^ and 2 × 10^5^ cells per well in triplicate and were maintained in complete medium to reach 70% of confluence on the next day. Cells were incubated at 37 °C in a 5% CO_2_ humidified atmosphere overnight. Transfection of RNP complex into Capan-2, MCF7 and T3M4 cells was next performed following the protocol entitled «Transfection of ssDNA donor oligonucleotides for HDR-mediated gene modifications using the Dharmacon™ Edit-R™ system» and using a lipid-based transfection reagent called DharmaFECT Duo (Dharmacon, Cat #T-2010-01). Briefly, a 2.5 µM Cas9 protein working solution, a 1 µM donor oligo working solution, a 2 µM gRNA transfection complex (with crRNA:tracrRNA) and a 6 µg/mL DharmaFECT Duo working solution were prepared from the stock solutions. RNP complex was then assembled with Cas9 protein at 25 nM, donor oligo at 10 nM and gRNA at 50 nM. The final transfection mixture was consisted of RNP complex, transfection reagent and serum-free medium, and was incubated 18 h under usual culture conditions. Two controls were also prepared: a gene editing control (RNP complex without donor oligo, CRISPR/Cas9 control (CC_ctrl)) and a negative control (non-transfected cells).

### Droplet digital PCR (ddPCR)

CRISPR/Cas9-mediated induction of mutations was controlled using droplet digital PCR (ddPCR). Briefly, total genomic DNA was extracted using DNeasy Blood and Tissue Kit (Qiagen, Hilden, Germany) according to manufacturer’s protocol for cultured cells. All DNA were quantified using Qubit 3.0 fluorometer (Life Technologies) and Qubit® dsDNA HS Assay Kit (Life Technologies).

Bespoken probes were designed to respectively detect *BRCA1* c.763G > T p.(E255*) (Bio-Rad, UniqueAssayID dHsaMDS901307380) and *BRCA2* c.2133C > A p.(C711*) mutations (Bio-Rad, UniqueAssayID dHsaMDS403398316). Reaction mix was prepared using 30 ng of DNA, 11 µL of 2X ddPCR supermix for Probes (no dUTP) (Bio-Rad, Hercules, CA, USA), 1 µL of ddPCR Mutation Assay (that contains primers and probes) and nuclease-free water to a final volume of 22 µL. DNA was replaced by 1 µL nuclease-free water for negative control. The ddPCR mix was loaded on a 96-wells reaction plate (DG32™ Cartridge, Bio-Rad) and placed into the QX200 Automated Droplet Generator (Bio-Rad) for droplets generation. Droplets were then transferred to a 96-wells PCR plate followed by PCR using the C1000 Touch™ Thermal Cycler (Bio-Rad) with the following conditions: 1 cycle for 10 min at 95 °C, 40 two-steps cycles (30 s at 94 °C and 60 s at 55 °C for *BRCA1* probe and 52.5 °C for *BRCA2* probe; ramp rate 2 °C/second), 1 cycle for 10 min at 98 °C and a 4 °C hold. The plate was finally analyzed using the QX200 Digital PCR Reader (Bio-Rad). Only samples with a number of droplets greater than 10,000 were analyzed according to manufacturer’s recommendations. DNA quantities (number of copies per microliter) obtained in the FAM channel (C_MUT_) and in the HEX channel (C_WT_) were determined using QuantaSoft analysis software version 1.7.4.0917 (BioRad) and the percentage of fractional abundance (FA) was calculated as follows: FA (%) = [C_MUT_ / (C_MUT_ + C_WT_)] × 100.

### Enzyme-linked immunosorbent assay (ELISA)

Sandwich enzyme-linked immunosorbent assays (ELISA) sandwich were performed to quantify BRCA proteins. Protein lysates were obtained using RIPA lysis buffer (Merck, Darmstadt, Germany) containing phenylmethylsulfonyl fluoride (PMSF) (Sigma-Aldrich) and centrifugation at 15,000 g for 20 min at 40 °C. The DCTM Protein Assay kit (Bio-Rad) was used for proteins quantification.

The ELISA kit for BRCA1 MSB2703253 (MyBioSource, San Diego, CA, USA) and the ELISA kit for BRCA2 MSB2703232 (MyBioSource) were used according to the manufacturer instructions. Briefly, 96-wells plates pre-coated with an antibody specific to BRCA1 or BRCA2 were provided, and 100 µL of diluted protein lysate were added into the appropriate wells with biotin-conjugated antibody specific to BRCA1 or BRCA2. Optical density (O.D.) was read at 450 nm wavelength using a Multiskan Ascent (Labsystems Titertek, Thermo Fisher Scientific). A standard curve for each protein was generated with known concentrations of the standard on the x-axis and corresponding absorbance (O.D. values on the y-axis using GraphPad Prism 9® (GraphPad Software, La Jolla, CA, USA). The line of best fit with an r^2^ value (coefficient of determination) ≥ 0.98 was selected.

### Crystal violet assay

Capan-2, MCF7 and T3M4 cells were respectively seeded into 96-wells plates at a density of 5 × 10^3^, 1 × 10^4^, and 2 × 10^5^ cells per well and were maintained in 200 µL of complete medium for 24 h. Cells were then exposed to 2, 5, 7, 10, 15, 25, 30, 40, and 50 µM concentrations of olaparib (Clinisciences, Nanterre, France) for 72 h. Olaparib solution stock was previously reconstituted in DMSO (Thermo Fisher Scientific, Waltham, MA, USA) according to the manufacturer’s instructions. Control cells were exposed to medium without olaparib or to 50 µM DMSO. After incubation, cells were fixed with 100 µL of 70% ethanol for 10 min. Next, 100 µL of 0.2% Crystal Violet (Sigma-Aldrich) in 20% ethanol was added into each well for 15 min, followed by four washes of distilled water and elution solution consisting of 100 µL of 0.1% acetic acid in 50% ethanol was added *per* well. Optical density was determined using a microplate reader (Multiskan Ascent; Thermo Fisher Scientific) at 540 nm. The half-maximal inhibitory concentration (IC50) values of olaparib were determined by a non-linear regression ([inhibitor] vs. normalized response) using GraphPad Prism 9® (GraphPad Software).

### Apoptosis detection assay

MCF7 and T3M4 cells were seeded into 6-wells plates at a density of 1 × 10^4^ cells/mL per well and Capan-2 cells at a density of 1 × 10^5^ cells/mL per well. Cells were maintained in complete medium for 24 h and then were exposed to 40 nM of olaparib or medium. After 72 h, 1 × 10^5^ cells were stained with annexin V/propidium iodide (PI) (BD Biosciences, Franklin Lakes, NJ, USA) according to the manufacturer’s instructions. Cell apoptosis was quantified within 1 h by flow cytometry using a Accuri C6Plus system (Becton Dickinson, San Jose, CA, USA).

### Statistical analysis

All experiments were performed in three independent tests of triplicates. Experimental data were analyzed by using one-way ANOVA or paired *t* test and differences were considered statistically significant when *P* value was less than 0.05. All graphs and statistical analysis were performed using GraphPad Prism 9®.

### Supplementary Information

Supplementary Table S1.

## Data Availability

All data generated or analyzed during this study are included in this published article and its supplementary information files.
