# Supplementary material for: CRISPR/Cas9-mediated knock-in of BRCA1/2 mutations restores response to olaparib in pancreatic cancer cell lines
Source: Sci Rep. 2023 Oct 31;13:18741. doi: 10.1038/s41598-023-45964-w (PMC10618219; doi:10.1038/s41598-023-45964-w)
Supplement: Supplementary file 1 — Supplementary Table S1. [file 41598_2023_45964_MOESM1_ESM.docx]

**Title**

**CRISPR/Cas9-mediated knockdown of *BRCA1/*2 restores response to olaparib in pancreatic cancer cell lines**

Andréa Witz^1,2,*^, Julie Dardare^1,2^, Aurélie Francois^2,3^, Marie Husson^1^, Marie Rouyer^1^, Jessica Demange^1^, Jean-Louis Merlin^1,2^, Pauline Gilson^1,2^, Alexandre Harlé^1,2^.

^1^Département de Biopathologie, Institut de Cancérologie de Lorraine, Vandœuvre-lès-Nancy, France.

^2^Université de Lorraine, CNRS CRAN UMR 7039, Vandœuvre-lès-Nancy, France.

^3^Département Recherche, Institut de Cancérologie de Lorraine, Vandœuvre-lès-Nancy, France.

*Corresponding author: Andréa Witz

Andréa Witz: a.witz@nancy.unicancer.fr

Julie Dardare: j.dardare@nancy.unicancer.fr

Aurélie Francois: a.francois@nancy.unicancer.fr

Marie Husson: m.husson@nancy.unicancer.fr

Marie Rouyer: m.rouyer@nancy.unicancer.fr

Jessica Demange: j.demange@nancy.unicancer.fr

Jean-Louis Merlin: [jl.merlin@nancy.unicancer.fr](mailto:jl.merlin@nancy.unicancer.fr)

Pauline Gilson: p.gilson@nancy.unicancer.fr

Alexandre Harlé: a.harle@nancy.unicancer.fr

Supplementary Table S1: Off-target sites predicted by the CrispRGold tool.

| **Rank** | **sgRNA sequence** | **Strand** | **Chr** | **Position** | **Annotation** |
| --- | --- | --- | --- | --- | --- |
| **1** | CTGAGAAGCGTGCAGCTGAGAGG | - | 64 | [43,094,766-43,094,785] | **BRCA1** |
|  | . . . . C . A . . . . . . . . . . . . G . . | - | 1 | [21,909,944 ; 21,909,965] | HSPG2, intron |
|  | . . . .^C^. . . . . . . . T . . . . . . G . . | + | 6 | [110,215,534 ; 110,215,556] | CDC40, intron |
|  | . . . . . . . T . . . . . . G . . . . . . . | + | 12 | [111,064,238 ; 111,064,259] | CUX2, intron |
| **2** | CTCAGCTGCACGCTTCTCAGTGG | + | 17 | [43,094,766-43,094,785] | **BRCA1** |
|  | . . . . . . - . . . . . . . . . . . .G . . | - | 1 | [17,674,602 – 17,674,622] | ARHGEF10L, intron |
|  | . . C . G . . . . . . . . . . . . . . A . . | - | 13 | [113,165,077 – 113,165,098] | PCID2 |
|  | . . .^C^. . . . . . G . . . . . . . . . A . . | + | 2 | [217,876,174 – 217,876,196] | TNS1, intron |
| **1** | TGATTCTCTGTCATGCCTGCAGG | + | 13 | [32,336,473 - 32,336,492] | **BRCA2** |
|  | . . G . G . . . . . . . . . . . . . . T . . | + | 16 | [81,382,708 – 81,382,729] | Intergenic |
|  | . . . . . . . . . C . . . . . . A . . . . . | - | 17 | [67,071,920 – 67,0717,941] | HELZ, 3UTR |
| **2** | TCTCTGTCATGCCTGCAGGAAGG | + | 13 | [32,336,477-32,336,496] | **BRCA2** |
|  | . . . . . . . . . A . . . . . T . . . . . . | + | 6 | [29,428,902 – 29,428,923] | OR5V1, intron |
|  | . . . . .^C^. . . . . T . . . . . . . . . . . | + | 14 | [36,007,239 – 36,007,261] | Intergenic |
|  | . . . . . . . . . . . . . . . . T A . G . . | + | 8 ; 13 | [130,746,763 – 130,746,784]  ; [95,371,119 – 95,317,140] | Intergenic |
|  | G . . . . C . . . . . . . . . . . A . G . . | - | 9 | [76,067,008 – 76,067,029] | PCSK5, intron |
|  | . A . . A . . . . A . . . . . . . . . . . . | - | Y | [21,077,553 – 21,077,574] | Intergenic |

Predicted off-target sites were given for both sgRNA sequence for the mutation BRCA1 G763T and for both sgRNAs for the mutation BRCA2 C711T. PAM sequences are colored in light blue.
